# Effect of Gamma Radiation on the Wear Potential of Hybrid Ceramic to Tooth Enamel

**DOI:** 10.3390/ma18030702

**Published:** 2025-02-05

**Authors:** Pollyanna Nogueira Ferreira da Silva, Fernanda Calvo Costa, Célio dos Santos Silva, Maria Carolina Barcellos, Sílvio Manea, Odair Lellis Gonçalez, Vitor Ribeiro Jardim, Gislene Valdete Martins, Nelson Lima, Anelyse Arata Found, Grace Mendonca De Souza, Rubens Nisie Tango

**Affiliations:** 1 Institute of Science and Technology, Sao Paulo State University—UNESP, Sao Jose dos Campos 12245-000, SP, Brazil; pollyanna.nogueira@unesp.br; 2 Department of Dental Materials and Prosthodontics, Institute of Science and Technology, Sao Paulo State University—UNESP, Sao Jose dos Campos 12245-000, SP, Brazil; fernanda.calvo@unesp.br (F.C.C.); celio.silva@unesp.br (C.d.S.S.); maria-carolina.barcellos@unesp.br (M.C.B.); 3 Space Engineering and Technology, National Institute for Space Research—INPE, Sao Jose dos Campos 12227-010, SP, Brazil; silvio.manea@inpe.br (S.M.); givmartins19@gmail.com (G.V.M.); 4 Department of Science and Aerospace Technology, Institute of Advanced Studies—IEAv/Brazilian Air Force, Sao Jose dos Campos 12228-001, SP, Brazil; odairlelisgoncalez@gmail.com; 5 Department of Lasers and Applications, Institute of Advanced Studies—IEAv/Brazilian Air Force, Sao Jose dos Campos 12228-001, SP, Brazil; vribeiro@ieav.cta.br; 6 Department of Materials Science and Technology, Nuclear and Energy Research Institute IPEN/CNEN-SP, Sao Paulo 05508-000, SP, Brazil; nblima@ipen.com; 7 Schulich School of Medicine & Dentistry, Western University, London, ON N6A 5C1, Canada; ane.arata@gmail.com; 8 School of Dentistry, University of Louisville, Louisville, KY 40202, USA; grace.desouza@louisville.edu

**Keywords:** radiotherapy, hybrid ceramic, materials properties

## Abstract

Hybrid ceramics exhibit low wear on antagonist tooth enamel, which may positively impact the oral rehabilitation of head-and-neck irradiated patients who experience alterations in tooth microstructure and wear resistance. This study aimed to evaluate the wear resistance of hybrid ceramics after gamma radiation exposure in contact with irradiated tooth enamel, as well as their mechanical and chemical properties. Notably, no previous studies focusing on the effects of radiation on hybrid ceramics were found in the literature. Vita Enamic discs and tooth fragments were subjected to daily doses of 2 Gy, totaling 0, 20, 40, 50, 60, and 70 Gy. The wear resistance of hybrid ceramics and a ceramic enamel analog (steatite) was tested against tooth enamel using a chewing simulation machine. Hybrid ceramic specimens underwent hardness, biaxial flexural strength, roughness, and FT-IR analyses. The data were analyzed using an ANOVA and Tukey’s test (α = 0.05). Enamic exposed to 60 and 70 Gy exhibited higher wear and caused less tooth enamel loss compared to steatite. The mechanical and chemical properties remained unchanged after irradiation. The roughness decreased across all groups after a chewing simulation but was not affected by irradiation. In conclusion, ionizing radiation did not alter the material’s properties but increased its wear.

## 1. Introduction

Ceramics are widely used in oral rehabilitation of oncologic patients because of their advantageous properties. They minimize the spread of ionizing radiation compared to metallic restorations, reducing the risk of secondary mucositis caused by radiation reflection [1]. Among ceramics, zirconia has been shown to undergo property changes under high doses of gamma irradiation [2]. However, hybrid ceramics have recently emerged as a promising alternative, although their behavior under ionizing radiation remains uninvestigated.

A porous feldspathic ceramic infiltrated with polymer under pressure and heat has been developed, resulting in a CAD/CAM block composed of 86% ceramic and 14% polymer [3,4,5]. This hybrid ceramic mimics the natural tooth structure because of its similar elastic modulus and hardness [3,6,7]. It also demonstrates distinct advantages, including lower wear on antagonist tooth enamel compared to other ceramic materials [8,9,10,11], as it is approximately four times harder than enamel [12] and exhibits unique crack propagation behavior [3,13].

Given its low wear potential on antagonist teeth and its mass loss comparable to that of tooth enamel [8], this material is considered a promising option for prosthetic rehabilitation in patients with head and neck cancer undergoing radiotherapy.

The choice of restorative material is critical, as gamma radiation has been shown to induce changes in dental structures depending on the radiation dose [14], including decreased scratch resistance [15], reduced enamel microhardness [14,15,16], weakened dentin–enamel junction strength [15,17,18], and increased surface roughness [14]. The enamel undergoes various changes following radiation exposure [19]. Radiation doses may increase the mineral density and decrease the mineral content [20]. Additionally, the organic matrix and protein content may decrease [21,22], micromorphological damage can occur [20,23], and the enamel’s hardness may reduce [21].

Despite this, no studies have evaluated the behavior of human tooth enamel subjected to simulated chewing against hybrid ceramics under gamma irradiation or the chemical–mechanical behavior of hybrid ceramics after irradiation.

This study aimed to address these gaps by testing the following hypotheses: (1) different doses of gamma irradiation increase the volume loss of hybrid ceramics during simulated chewing against tooth enamel, and (2) ionizing radiation causes alterations in the mechanical (hardness and biaxial flexural strength) and chemical properties of hybrid ceramics. Remarkably, no studies were found in the literature focusing specifically on the effects of radiation on hybrid ceramics opposed to tooth enamel. The evaluation of ceramic properties is essential to guide dental professionals in selecting restorative materials that are both satisfactory and viable for patients undergoing radiotherapy. This ensures that materials meet the specific needs of these cases. Additionally, this assessment can aid in diagnosing patients who have already received ceramic restorations and were subsequently exposed to radiation, as oral complications following cancer therapy are common and can negatively affect quality of life [19].

## 2. Materials and Methods

Pin-shaped specimens (4 mm in diameter × 15 mm in height, with a 2 mm chamfer) [18,24] of hybrid ceramic (Vita Enamic, Vita Zahnfabrik, Bad Säckingen, Germany) and enamel analog ceramic (steatite, Chiarotti Ceramics, Jaguariúna, SP, Brazil) were prepared from commercially available materials.

Sixty third molars, freshly extracted for orthodontic reasons from patients aged 15-25 years (approved by the Brazilian Platform, protocol CAAE 66495417.1.0000.007) were cleaned and stored frozen in 2% chloramine solution for no longer than six months. The teeth were sectioned buccolingually and mesiodistally using a diamond wheel (Extec High Concentration, Extec, Enfield, CT, USA) under water irrigation with a precision cutter (IsoMet 1000 Precision Saw, Buehler, Lake Bluff, IL, USA), resulting in four crown fragments per tooth. The fragments were randomly assigned on 3 December 2018 (www.random.org) to groups according to the gamma radiation dose (n = 10): control (0 Gy), 20 Gy, 40 Gy, 50 Gy, 60 Gy, and 70 Gy. Fragments were embedded in auto-cured acrylic resin, exposing their buccal or lingual surfaces, which were sequentially flattened with #1200, 2500, and 4000 grit SiC sandpapers. Irradiation was performed using a cobalt-60 teletherapy radiator (Eldorado 78, Atomic Energy of Canadian Limited, Chalk River, ON, Canada) at a daily dose of 2 Gy, 5 days per week, with doses controlled using a dosimeter (Radiation Monitor Controller, Model 2026C, Monrovia, CA, USA). Between irradiation sessions, specimens and fragments were stored in distilled water.

### 2.1. Chewing Simulation

A pin of each material and a tooth fragment were mounted as antagonists in a pin-on-block physiological wear test machine (Biocycle V2, Biopdi, São Carlos, SP, Brazil), with the following parameters: 20 N, 400,000 cycles [8], 1.7 Hz [16], horizontal occlusal-cervical movement of 2 mm, immersed in distilled water.

### 2.2. Optical Profilometry

Specimens were scanned using a confocal microscope (CyberSCAN CT 100, Cyber Technologies GmbH, Ingolstadt, Germany) with a 3 mm lens, exposure time of 5000 ms/0.25 ms for a 20 μm step. For tooth fragments, the exposure time was 2000 ms/0.25 ms with a 10 μm step. Three roughness measurements per specimen (n = 10) were taken to calculate the average roughness (Ra, μm) and volume loss (mm³) of the material pins and tooth enamel. Epoxy resin replicas of the tooth fragments and specimens were sputter-coated with gold using an ion sputter coater (SC7620 ‘Mini’ Sputter Coater/Glow Discharge System, EMITECH, East Sussex, UK). Topography was analyzed with a scanning electron microscope (SEM; Inspect S 50, FEI Company, Brno, Czech Republic) at 15–25 kV, 5.0 spot size, and magnifications of 75× for replicas and 65× for pins.

### 2.3. Production of Ceramic Discs and Tests

Vita Enamic blocks (n = 50) were cut with a 15 mm diamond drill (Small Tools, Belenzinho, São Paulo, Brazil) and sectioned using a diamond wheel (Extec High Concentration) mounted on a cutting machine (IsoMet) to obtain specimens with final dimensions of 12 mm in diameter × 1.2 mm thick (ISO 6872 [25]). The specimens were sequentially polished with #400-, #600-, and #1200-grit SiC sandpapers on a polishing machine (EcoMet 250, Buehler, Uzwil, Switzerland) and then randomly divided into groups based on the radiation dose (n = 10): control (0 Gy), 20 Gy, 40 Gy, and 70 Gy. Irradiation followed the same procedure as described in Section 2.

### 2.4. Biaxial Flexural Strength Test

Flexural strength was measured using a biaxial flexural test (ISO 6872) in a universal testing machine (EMIC DL-1000, EMIC, São José dos Pinhais, PR, Brazil). Specimens were loaded at the center with a rounded chisel (3 mm radius) at a crosshead speed of 1 mm/min until fracture (n = 10). Flexural strength (σ) was calculated using the following formula: σ¼3Pl/2wb2, where P is the fracture load (N), l is the support span (12 mm), w is the specimen width (mm), and b is the specimen thickness (mm).

### 2.5. Hardness and Elastic Moduli

Four additional specimens per group were polished sequentially with #1200-, #2400-, and #4000-grit SiC sandpapers. A Berkovich penetrator coupled to a nanoindenter (NHT2, Anton Paar, Graz, Austria) was used to measure the hardness and elastic moduli. The test parameters included a 10 Hz frequency, 25 mN load for 10 s, and a loading/unloading rate of 50 mN/min, with three measurements per specimen.

### 2.6. FT-IR

Two specimens from each group underwent Fourier-transform infrared spectroscopy (FT-IR; Perkin Elmer Spectrum One Fourier Transform Infrared Spectrometer, Waltham, MA, USA) using the UATR technique, with a transmission range of 4000–650 cm^−1^.

### 2.7. Statistical Analysis

Data on the tooth volume loss against the Enamic and steatite (mm^3^), Enamic and steatite volume loss (mm^3^), roughness (Ra), flexural strength (MPa), and hardness (VHN) were analyzed using ANOVA and Tukey’s test, with a significance level of α = 0.05 (JASP, Amsterdam, The Netherlands).

## 3. Results

The results obtained from the analyses were organized to address the research questions proposed in this study. The evaluations included parameters such as volumetric loss, surface roughness, flexural strength, hardness, and elastic modulus, as well as topographical and spectroscopic analyses of the materials. The data presented were statistically analyzed to determine the significance of the differences between the experimental groups exposed to varying radiation doses and the control groups.

The ANOVA revealed a significant interaction between material type and irradiation dose on the tooth volume loss. Tooth volume loss increased progressively with higher irradiation doses, with the greatest loss observed at 60 Gy and 70 Gy (Table 1).

**Figure 1:**
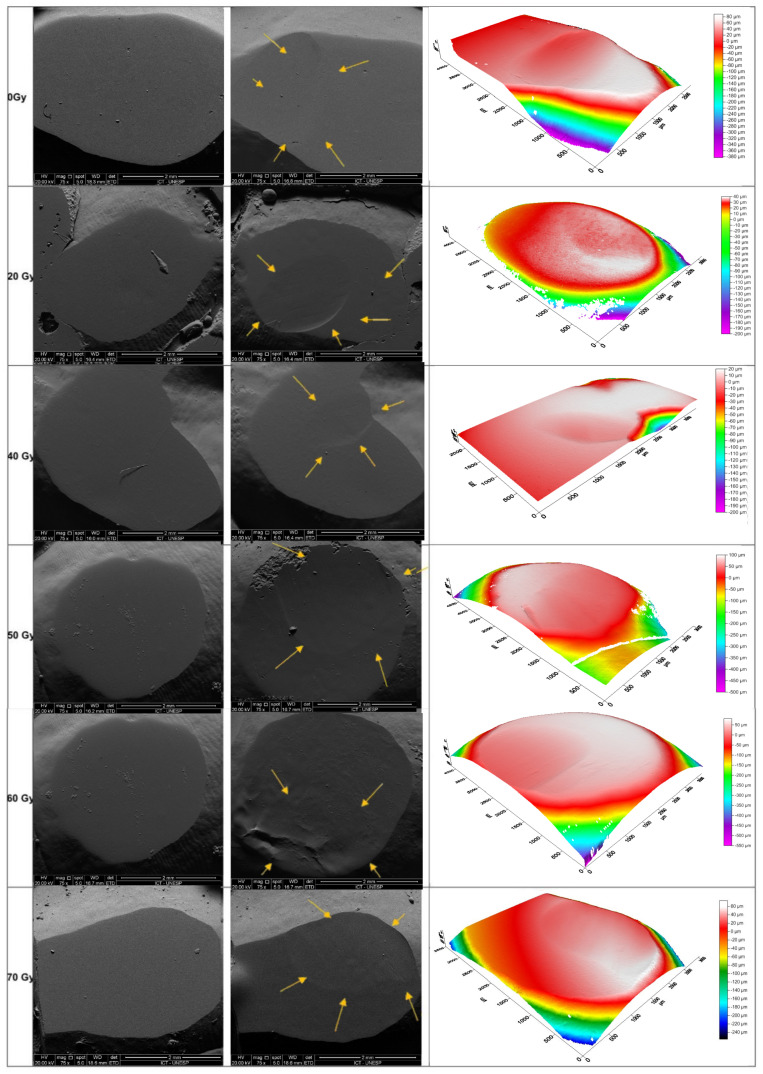
SEM images and optical profilometry of tooth enamel before and after the chewing simulation against the Enamic. Key: SEM Images (75× magnification): control group; group irradiated with 20 Gy; group irradiated with 40 Gy; group irradiated with 50 Gy; group irradiated with 60 Gy; group irradiated with 70 Gy. The yellow arrows indicate the path of the wear.

**Figure 2:**
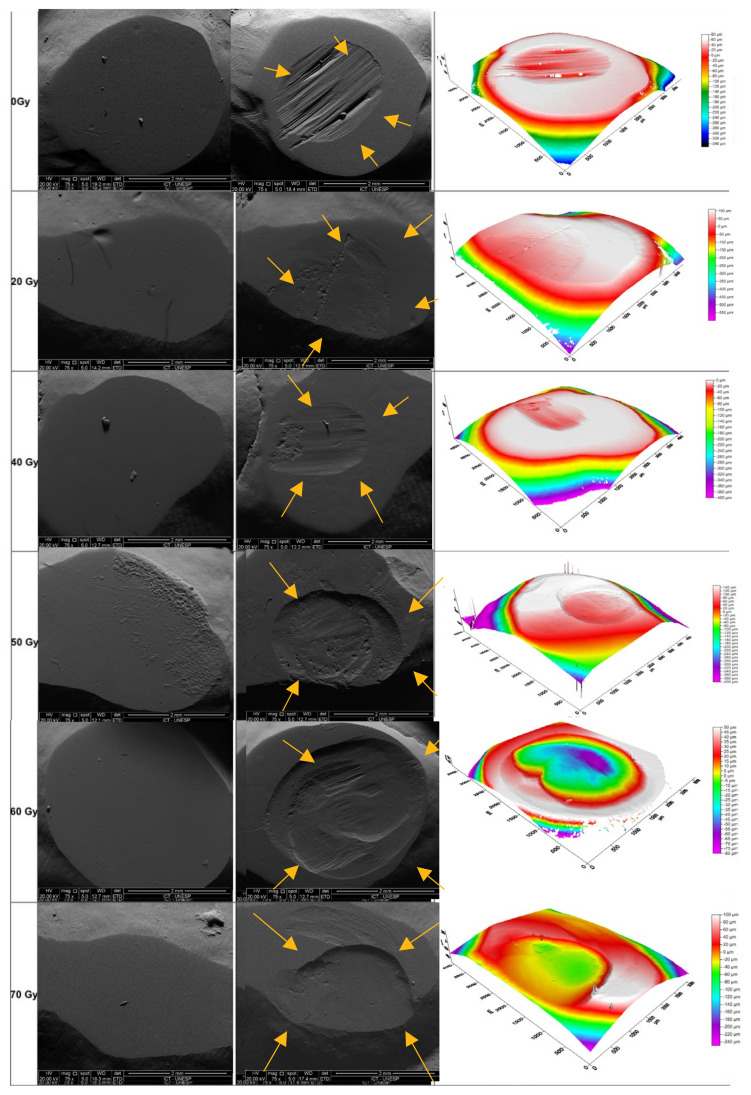
SEM images and optical profilometry of tooth enamel before and after the chewing simulation against the steatite. Key: SEM Images (75× magnification): control Group; group irradiated with 20 Gy; group irradiated with 40 Gy; group irradiated with 50 Gy; group irradiated with 60 Gy; group irradiated with 70 Gy. The yellow arrows indicate the path of wear.

Figure 1 and Figure 2 presents SEM images (75× magnification) and optical profilometry of tooth enamel following the chewing simulation against the Enamic and steatite, respectively.

Enamic exhibited greater volume loss compared to steatite (Table 2).

The roughness of both materials decreased after the chewing simulation, and Enamic exhibited lower nanohardness and elastic modulus compared to steatite (Table 3).

**Figure 3:**
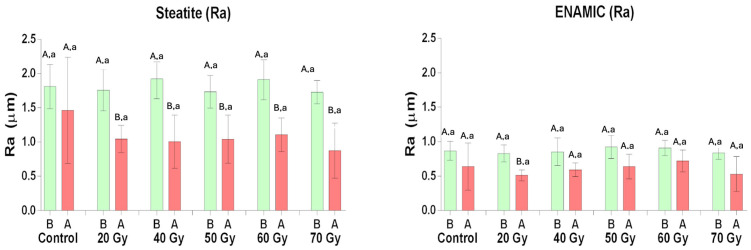
Column graph (mean ± SD) of Ra (μm) according to the groups. Different uppercase letters represent significant differences in the Ra between the baseline and after the chewing simulation, and different lowercase letters represent differences between the irradiation doses.

Figure 3 shows the results of the roughness test (Ra). There was a decrease in the roughness values for all groups after the chewing simulation, except for the control group (*p* < 0.005).

**Figure 4:**
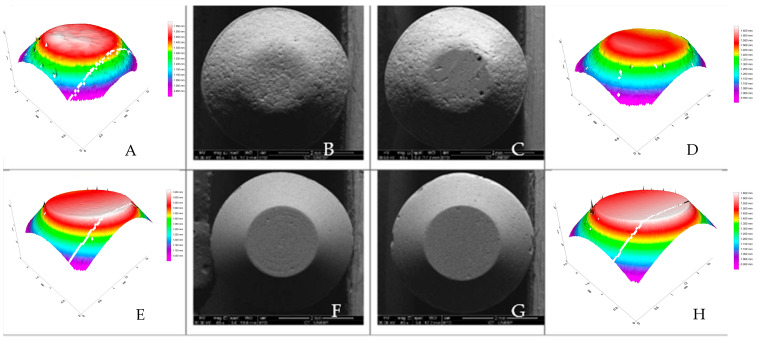
SEM (65× magnification) and optical profilometry images of the Enamic and steatite pins. Key: SEM Images (65× magnification): (**A**,**B**) are baseline images of the steatite; (**C**,**D**) are images of the steatite after the chewing simulation; (**E**,**F**) are baseline images of the Enamic; (**G**,**H**) are images of the Enamic after the chewing simulation.

Key: SEM and optical profilometry images reveal a smoother, more regular surface for the Enamic compared to the steatite, both at baseline and after the chewing simulation (Figure 4). Different uppercase letters represent significant differences in the Ra between the baseline and after the chewing simulation, and different lowercase letters represent differences between the irradiation doses.

There were no changes in the flexural strength and hardness of the Enamic with the various irradiation doses (Table 4).

**Figure 5:**
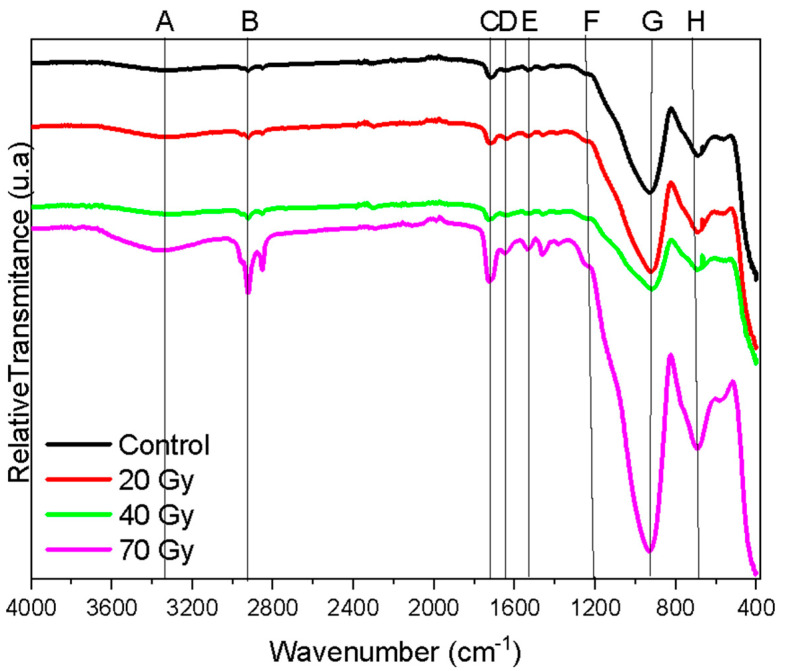
FT-IR spectra (UATR) of the Enamic hybrid ceramics.

Figure 5 shows the FT-IR spectra (UATR) of the Enamic at different irradiation doses (uppercase letters represent the band groups listed in Table 5). The Enamic exhibits inorganic components corresponding to bands F, G, H, tetrahedral aluminum, and tetrahedral silica, while the organic components are related to the polymer constituting the ceramic material. Regarding each band, A corresponds to the stretching of N-H bonds; B is attributed to the symmetrical and asymmetrical C-H bonds of the carboxylic groups; C and D are carbonyl bands associated with the polymeric network of urethane dimethacrylate (UDMA) and triethylene glycol dimethacrylate (TEGDMA).

## 4. Discussion

In the present study, the mechanical properties and chemical composition of the hybrid ceramic were not altered by gamma irradiation, and it exhibited a low wear capacity against tooth enamel. This characteristic of low wear against dental enamel has been previously reported [8,10,11,19,20,21,22,23]. However, this is the first study to evaluate this property in the context of ionizing radiation.

In comparison with other restorative materials, teeth restored with either flowable resin composite or glass ionomer cement exhibited decreased surface hardness under 60 Gy [26] and 70 Gy [19] radiation. No changes were observed in the compression test and compressive strength between these materials [19]. Interestingly, the microhardness of the resin composites increased when subjected to radiation [27]. Compared to these findings, hybrid ceramics demonstrated superior performance in the present study; hence, there were no changes in the material properties upon radiation. The findings of the present study align with the existing literature, which indicates that even under high doses of radiation, the composite structure of ceramics remains unchanged [28].

The low wear capacity of the hybrid ceramic is considered advantageous, as tooth enamel exposed to 60 Gy and 70 Gy doses exhibited greater volume loss compared to the other groups (Table 1 and Figure 1). This susceptibility is likely due to an increased carbonate/phosphate ratio, which reduces the enamel hardness [15,16], degradation of types IV and VII collagen responsible for anchoring enamel to dentin [24], and a reduction in scratch resistance [15]. Most in vitro studies apply a single dose of 60 Gy, although the dose depends on the tumor’s type, size, and location. In this study, different doses were applied to better simulate a realistic radiotherapy regimen [29].

The low wear on tooth enamel by Enamic during radiotherapy (Figure 2 and Table 1) may be explained by its lower surface roughness and hardness [8,11,28,30,31,32], compared to steatite (Table 3, Figure 3 and Figure 4). Increased surface roughness is associated with greater wear of the antagonist material [11,33,34], and hybrid ceramics are more easily machined, resulting in a smoother and more uniform surface [35]. Therefore, hybrid ceramics could be considered a good option for prosthetic tooth rehabilitation in head-and-neck irradiated patients. Other ceramic materials have shown changes in their properties when exposed to low doses of ionizing radiation [36,37,38].

Although Enamic consists of a porous feldspathic ceramic matrix infiltrated with a polymer [3,4,5], it demonstrated similar behavior to other ceramics, such as zirconia [37], when exposed to ionizing radiation, with no changes in the mechanical properties (Table 4).

While ionizing radiation can induce an additional degree of polymerization [38], potentially increasing the hardness [39,40], no significant increase in the hardness values was observed in this study (Figure 5 and Table 5). Within the limitations of this in vitro study, we are able to confirm the stability of Enamic’s compositional and mechanical properties under ionizing radiation, resulting in lower wear of tooth enamel. The sharpening of the digital x-ray band peak (Figure 5) can be due to the deformation of enamel hydroxyapatite crystal; however, the shape factor of the enamel was not tested in the present study [41].

Notably, although this is the first study to evaluate the properties of hybrid ceramics in comparison to tooth enamel following radiation, it also presents limitations. The main limitation of this study lies in the absence of long-term evaluation tests, assessments of the effects of combined thermomechanical aging, and randomized clinical trials to evaluate the wear patterns of this material and the antagonist tooth enamel. These analyses are crucial for simulating more realistic oral conditions. The promising results pave the way for new studies that can further explore these findings while addressing the identified limitations. Therefore, future research should aim to include these aspects to build upon the findings of the present study.

## 5. Conclusions

Limited by the methodology employed, it can be concluded that:

- The mechanical and chemical properties of the Enamic remained unchanged after exposure to gamma radiation, even at doses as high as 70 Gy;
- Enamic demonstrated a lower wear resistance and a reduced capacity to wear the antagonist tooth enamel compared to steatite, across various doses of gamma radiation.

## Figures and Tables

**Table 1:** Tukey’s test results for the comparison of the irradiated tooth volume loss (mm^3^) during a chewing simulation against Enamic and steatite. Different uppercase letters in the columns represent significant differences (*p* = 0.000).

Material	Radiation	Mean/Standard Deviation
Enamic	70 Gy	0.08 ± 0.03 ^C^
	60 Gy	0.06 ± 0.04 ^C^
	50 Gy	0.05 ± 0.03 ^C^
	40 Gy	0.04 ± 0.01 ^C^
	20 Gy	0.05 ± 0.01 ^C^
	0 Gy	0.05 ± 0.02 ^C^
Steatite	70 Gy	0.77 ± 0.24 ^A^
	60 Gy	0.74 ± 0.51 ^A^
	50 Gy	0.41 ± 0.23 ^B^
	40 Gy	0.25 ± 0.06 ^BC^
	20 Gy	0.23 ± 0.17 ^BC^
	0 Gy	0.23 ± 0.14 ^BC^

**Table 2:** Results of Tukey’s test for the comparison of volume loss (mm^3^) between Enamic and steatite. Different uppercase letters in the column indicate significant differences.

Material	Mean/Standard Deviation
Enamic	0.36 ± 0.19 ^A^
Steatite	0.04 ± 0.02 ^B^

**Table 3:** Results of the mean roughness (Ra-μm) before and after the chewing simulation, nanohardness (VHN), and elastic modulus (GPa) of the materials. Different uppercase letters in the columns represent significant differences.

Material	Baseline Roughness	Upon Chewing Roughness	Nano Hardness	Modulus of Elasticity
Enamic Steatite	0.8 ± 0.1 ^A^ 1.8 ± 0.2 ^B^	0.3 ± 0.2 ^A^ 0.7 ± 0.3 ^B^	413.7 ± 125.7 913.5 ± 96.4	37.049 ± 4.3 95.61 ± 15.5

**Table 4:** Results for the flexural strength (MPa) and hardness (HV) of the Enamic at various irradiation doses. Different uppercase letters in the columns represent significant differences.

Dose	Flexural Strength (MPa)	Hardness (VHN)
Control (0 Gy)	108.82 ± 12.68 ^A^	413.73 ± 125.70 ^A^
20 Gy	107.90 ± 10.65 ^A^	408.04 ± 134.62 ^A^
40 Gy	104.81 ± 11.76 ^A^	426.85 ± 122.54 ^A^
70 Gy	102.43 ± 14.92 ^A^	417.51 ± 191.45 ^A^

**Table 5:** Table of the band set assignments shown in Figure 5.

Frequencies (cm^−1^)	Assignment	Band Set	Reference
≈3432	NH/-OH	A	Ramos et al., 2016 [4]
≈2912	C-H	B	Ramos et al., 2016 [4]
1730	C=O	C	Ramos et al., 2016 [4]
1617	C=O	D	Ramos et al., 2016 [4]
1530	Amide	E	Ramos et al., 2016 [4]
1070	Si-O-Si/Si-O-Al	F	Ramos et al., 2016 [4]
1058	Si-O-Si/Si-O-Al	G	Ramos et al., 2016 [4]
792	Al-O	H	Ramos et al., 2016 [4]

## Data Availability

The original contributions presented in this study are included in the article. Further inquiries can be directed to the corresponding author.
